# Sepsis-induced Atrial Fibrillation: Can We Predict and Prevent This High-Risk Complication?

**DOI:** 10.7759/cureus.85387

**Published:** 2025-06-05

**Authors:** Vahagn Tamazyan, Aleksan Khachatryan, Ashot Batikyan, Hakob Harutyunyan, Binit Aryal, Supraja Achuthanandan, Gerald Hollander

**Affiliations:** 1 Department of Internal Medicine, Maimonides Medical Center, Brooklyn, USA; 2 Department of Cardiovascular Medicine, Icahn School of Medicine at Mount Sinai, Mount Sinai Hospital, New York, USA; 3 Department of Internal Medicine, Albert Einstein College of Medicine, Jacobi Medical Center, North Central Bronx Hospital, New York, USA; 4 Department of Cardiology, Maimonides Medical Center, Brooklyn, USA

**Keywords:** anticoagulation, atrial fibrillation, critical illness, inflammation, prediction models, prevention, sepsis, stroke risk

## Abstract

Sepsis-induced new-onset atrial fibrillation (NOAF) is a frequent and serious complication in critically ill patients, linked to increased morbidity, mortality, stroke, and prolonged hospitalization. Though often transient during acute illness, NOAF is now recognized as a predictor of long-term cardiovascular vulnerability, with recurrence rates exceeding 50% over five years and a significantly heightened risk of thromboembolic events. Stroke risk is notably elevated in this population, yet the role of anticoagulation remains uncertain due to potential bleeding complications and lack of clear guidelines. This review consolidates current evidence on the pathophysiology, incidence, risk factors, and clinical impact of sepsis-induced NOAF, with a focus on stroke prevention, recurrence, anticoagulation challenges, and emerging predictive models. Additionally, it examines prevention strategies that target inflammatory and hemodynamic pathways.

## Introduction and background

Sepsis-induced new-onset atrial fibrillation (NOAF) is a common and clinically significant complication in critically ill patients, contributing to increased morbidity, mortality, and prolonged hospital stays. It is typically defined as atrial fibrillation (AF) occurring for the first time during sepsis or within seven days of its onset, confirmed via ECG, and without a prior history of AF [1-3]. The proarrhythmic environment in sepsis is driven by systemic inflammation, autonomic dysfunction, electrolyte imbalances, and myocardial stress, making septic patients highly susceptible to AF [4-9]. Although NOAF in sepsis is often transient, it is associated with a higher risk of hemodynamic instability, thromboembolic events, and poor long-term cardiovascular outcomes, serving as a marker of poor prognosis [2,10,11]. For instance, recurrence rates have been reported to exceed 50% over five years of follow-up in some observational cohorts. [12-14]. Stroke and anticoagulation deserve particular attention, as patients with NOAF face a significantly increased risk of stroke. However, the benefits of anticoagulation in this setting remain uncertain and are complicated by concerns about bleeding.

Although interest in predictive models and preventive strategies is growing, these tools are still underused in clinical practice. This review provides a comprehensive evaluation of the evidence on sepsis-induced NOAF, focusing on its pathophysiology, risk factors, clinical outcomes - particularly stroke risk and anticoagulation - and recurrence, prediction models, prevention, and management strategies.

## Review

Pathophysiology

Inflammation plays a central role in the development of NOAF during sepsis. Pro-inflammatory cytokines such as interleukin-6 (IL-6), tumor necrosis factor-alpha (TNF-α), and interleukin-1 (IL-1) drive a cytokine storm that affects cardiac tissue, leading to atrial fibrosis, altered electrophysiology, and increased arrhythmogenesis [4.5]. Oxidative stress, triggered by this inflammatory response, further contributes to atrial remodeling and fibrosis, destabilizing the heart's electrical activity [6]. The hyper-inflammatory state of sepsis, combined with impaired myocardial function and aggressive fluid resuscitation, can elevate left ventricular end-diastolic pressure, causing left atrial stretch, a key substrate for AF. Also, sepsis-induced ventricular remodeling reduces ventricular compliance, disrupting left atrial and pulmonary venous hemodynamics, thereby amplifying arrhythmogenic potential [10]. Autonomic dysfunction during sepsis is another major contributor to AF. The increased sympathetic activation during the stress response increases heart rate and myocardial contractility, making the heart more susceptible to arrhythmias. At the same time, vagal tone suppression eliminates its protective effects against AF, further increasing the risk [7,8]. Additionally, myocardial injury and electrolyte imbalances, such as hypokalemia and hypomagnesemia, are common in septic patients and disrupt cardiac action potentials, contributing to AF onset [9,15]. Experimental studies support this, with a guinea pig model showing that sepsis significantly shortens atrial action potential duration (APD) due to nitric oxide (NO)-mediated ion channel dysfunction, creating an electrophysiological environment prone to AF [16].

Incidence and mortality

NOAF is a common complication in critically ill patients with sepsis, with an overall incidence of 23% in intensive care unit (ICU) settings. AF risk escalates with sepsis severity, affecting 10% of sepsis cases, 22% of severe sepsis cases, and up to 46% of septic shock patients. Patients with severe sepsis face a sevenfold increased AF risk compared to non-septic individuals, while septic shock increases AF risk 10-fold. Most AF episodes occur within the first 36 hours of ICU admission [1-3]. Continuous ECG monitoring in septic shock patients has shown that 34% experience silent AF, which often goes undetected but carries the same poor prognosis as symptomatic AF [17] . NOAF during sepsis is consistently associated with significantly higher in-hospital mortality and worse outcomes. Walkey et al. reported an in-hospital mortality rate of 56% for patients with NOAF during severe sepsis, exceeding the rates for those with pre-existing AF (44%) and without AF (38%). This study also demonstrated a 7% increased adjusted risk of death, suggesting NOAF is a grave prognostic marker, potentially contributing to mortality through heart failure, stroke, or refractory hypotension [2]. Likewise, Gandhi et al. associated NOAF in sepsis with increased morbidity, ICU and hospital length of stays, and risk of stroke [10]. In further support of the association was Xiao et al.'s meta-analysis of 225,841 patients, which also showed a confirmed increase in risk, indicating that NOAF in sepsis has a 2.09 times increase in in-hospital mortality as well as a 2.44 times increase in post-discharge mortality in comparison to those without AF [11]. These findings established that NOAF is not merely a self-limiting arrhythmia but a prognostic indicator.

Risk factors

Studies have also identified significant risk factors for developing NOAF during sepsis, such as patient-related factors (age, underlying condition) and sepsis-related factors (organ dysfunction, source of infection, therapeutic interventions). Bosch et al. [18] evaluated 44 potential risk factors for the condition of NOAF during sepsis. A meta-analysis of a pool of 18 factors found seven factors posing a significant risk, such as advanced age, acute organ failure, obesity, endocarditis/bacteremia, acute kidney failure, prior congestive heart failure (CHF), and right heart catheterization. Right heart catheterization and CHF provided the most significant risk increase, over 50%. Five factors, on the other hand, lessened the risk of new-onset AF: other races (black) compared to race (white), diabetes mellitus, urinary source of infection, and corticosteroid therapy, with the most pronounced risk reduction being by corticosteroids, over 50%. Six factors - hypertension, female gender, acute respiratory failure, gastrointestinal, respiratory, and cutaneous origins of infection - were not significantly related to NOAF. Qualitative analysis brought forth further risk factors from individual studies, such as fungal infection, prior stroke, ICU admission, and hypotension requiring vasopressors, which raised the risk. Conversely, high albumin levels and hospital admission to a medical ICU (compared to a surgical ICU) lowered the risk. Sepsis-specific determinants such as fungal infection, vasopressor requirement, and high inflammatory markers (e.g., high levels of C-reactive protein (CRP)) had a more significant role in developing NOAF in sepsis. These findings warrant further research and external validation to better understand their clinical relevance [18]. According to a study by Zakynthinos et al. on the topic of NOAF occurring in intubated ICU-COVID-19 with ARDS patients, sepsis and subsequent secondary bacterial infections were determined to be the primary stimuli for NOAF in critically ill COVID-19 patients. A total of about 84.2% of the episodes of NOAF occurred concomitantly with septic episodes complicated by septic shock [19]. In an extensive study of over 1.4 million sepsis subjects, Liu et al. determined the main risk factors for NOAF by identifying geriatric age (>80 years) as having a sixfold higher incidence than the 18- to 65-year age cohort. Older age as a risk factor was confirmed by multivariable analysis as the highest, followed by concomitant cardiovascular diseases such as congestive heart failure, myocardial infarction, coronary disease, and necessity for mechanical ventilation. Those necessitating renal replacement therapy or septic shock were also considered at risk [20]. Another investigation of risk factors and infectious pathogens in sepsis patients with NOAF reported that *Pseudomonas aeruginosa*, *Streptococcus pneumonia*, and central venous catheters were independent predictors of its occurrence [21].

Recurrence of AF following sepsis

Sepsis-induced NOAF has a high risk of recurrence, with all studies reporting over 50% of recurrences and 61% of those with infection-induced AF recur [12]. At five years of follow-up, 55% recur [13]. During the initial year following sepsis, 36% are hospitalized for a recurrence of AF, and the condition is associated with a doubling of thromboembolic events, such as stroke [14]. These observations suggest that spells of AF in sepsis are not benign or self-limiting but must be aggressively treated on a par with non-infection-related AF. Sepsis-induced AF is a sign of an ongoing cardiovascular vulnerability that necessitates careful long-term follow-up and aggressive management strategies. Individuals recovering from sepsis-induced AF may need extensive care, such as anticoagulation therapy, antiarrhythmic drugs, and heart failure management. Long-term follow-up is achieved by prioritizing regular cardiac examinations, monitoring for AF recurrences, optimizing cardiovascular risk factors, and individualized treatment based on underlying comorbidities.

Risk of stroke and use of anticoagulation in sepsis-induced AF

Sepsis-induced NOAF considerably raises short- and long-term stroke risk compared with pre-existing AF or non-AF patients. During hospital stay, the incidence of ischemic stroke is as high as 2.6% in patients with sepsis-induced NOAF, compared with 0.57% in those with pre-existing AF. The development of sepsis-induced AF raises the risk of in-hospital stroke above that of sepsis patients with no AF and is associated with more significant in-hospital mortality [2]. Stroke risk persists well beyond the acute setting [22]. In five years, patients with sepsis-induced AF remain at a significantly raised ischemic risk of stroke comparable to other types of AF [12,13]; the initial two years following sepsis are particularly worrisome, with several studies noting a 6.6-fold increase in the risk of ischemic stroke within two years for survivors with NOAF. Partially offsetting the raised risk of sepsis-induced AF for stroke, anticoagulation effectiveness remains unclear, driven primarily by bleeding concerns. In a trial of 35,500 subjects, 35% were given parenteral anticoagulants with no reduction in ischemic stroke observed, however, at the cost of rising danger of bleeding [22-24]. Quon et al. also reported no substantial reduction of stroke with anticoagulation in the setting of new-onset secondary AF. The study did, however, underscore higher bleeding risks, particularly among those with acute pulmonary disease. Other studies also indicate that anticoagulants are given to those who are at high risk of stroke increasingly often. However, with no anticipated reduction in stroke, discontinuation or re-administration of anticoagulants continues to be widespread following discharge [25,26]. A meta-analysis of randomized controlled trials (RCTs) assessing anticoagulant use in sepsis did not find significant survival benefits. However, it confirmed raised bleeding complication risk in all patient subsets [27]. Stroke risk estimation is also a problem. The CHA₂DS₂-VASc score, widely used for AF-related stroke risk, has shown poor predictive value in sepsis-induced AF, and alternative models have demonstrated only modest accuracy [25,28]. The effectiveness of anticoagulation at reducing the risk of stroke in sepsis-induced AF is unclear, even though it is effective in reducing the risk of stroke in non-sepsis-induced AF patients. Anticoagulation during sepsis had no significant reduction of stroke risk, with a raised risk of bleeding. This implies that the risks may exceed the benefits of anticoagulation in certain sepsis patients. The absence of a clinically significant reduction in the risk of stroke with anticoagulation implies that sepsis-related AF may involve novel mechanisms of thrombosis formation different from classical thromboembolism originating from the left atrium. Non-cardioembolic mechanisms such as hemodynamic instability related to the imposition of AF on sepsis could be responsible for the generation of a low-flow state leading to stroke. This remains a hypothesis and requires further investigation to understand the underlying mechanisms. The CHA₂DS₂-VASc score, used in assessing the long-term risk of stroke in AF, has limited applicability in sepsis-induced AF. The score is used to predict the risk of a one-year stroke, with the actual thromboembolic risk of stroke during hospitalization probably being very low. Therefore, anticoagulation at early stages may provide minimal benefit in the prevention of stroke during sepsis-induced AF. Furthermore, those with sepsis or septic shock may need to undergo invasive intervention, such as surgery, dialysis, or central venous catheterization, which carries a high risk of bleeding. Standard instruments of risk assessment may not serve these complexities well. Management of sepsis-induced AF must be better studied to determine the right anticoagulation strategy, weigh prevention of stroke against bleeding risks, develop new instruments to assess for stroke, and account for sepsis's built-in inflammatory as well as coagulopathic state.

Prediction

Prediction of NOAF in septic patients allows the clinician to modulate treatment strategies and eschew precipitating factors. The models of NOAF prediction help in the formation of preventive measures to curb the occurrence of AF, improve the patient's survival, and avoid complications. The Atrial Fibrillation in Sepsis (SAFE) score by Klein Klouwenberg et al. [1] was developed to predict the risk of occurrence of the development of NOAF in critically ill sepsis ICU patients. The tool emerged from a study attempting to demystify AF's occurrence, predictors, and outcome in such a high-risk group. SAFE score predicts the occurrence of AF in the first seven days of ICU admission, a particularly early, reliable risk assessment parameter [1]. One of the score's most remarkable qualities is the simplicity of using generally available variables. The SAFE score can be used in various healthcare settings and is available in everyday clinical practice. The SAFE score utilized several demographic and clinical factors that are known to correlate with the onset of AF in sepsis. These include age, body mass index (BMI), immunocompromised status, and markers of illness severity, for example, septic shock status and vasopressor or inotrope utilization. It also involves inflammatory markers such as CRP value and white blood cell count (WBC), renal failure status, potassium level, and fraction of inspired oxygen (FiO_2_). The SAFE score has shown good discrimination with a C-statistic of 0.81, thus indicating superior discriminatory value in classifying at-risk subjects with AF. High discrimination implies that the score can confidently discriminate between the likeliness and the unlikeliness to develop AF. The model was also well-calibrated such that the estimated risk approximated the observed proportions of AF, making it a good tool for clinical management in the ICU [1]. In a study of external validation by Rucci et al., the SAFE score's performance was tested in a critically ill sepsis patient cohort. The original model performed poorly in the new patient group, with suboptimal discrimination and low accuracy. Following the modification of the model, its accuracy improved along with its calibration. Even after such modification, however, the model had a low positive predictive value (PPV), which means it was not always successful in identifying those that eventually developed AF [29]. Long et al. found a high association of early disorders of coagulation with an elevated risk of the occurrence of AF among septic patients. According to their findings, sepsis patients who show evidence of early coagulation abnormalities, characterized by changes in the platelet count, elevated international normalized ratio (INR), and prolonged activated partial thromboplastin time (APTT), are at a considerably higher risk of developing AF [30]. Li Z et al. [31] created and validated a nomogram model predicting new-onset AF (AF) in sepsis based on clinical information from 2,492 sepsis patients from two centers. The model selected independent predictors of NOAF, such as age, INR, fibrinogen, CRP, Sequential Organ Failure Assessment (SOFA) score, CHF, and the use of dopamine. The model had superior discrimination, calibration, and clinical usefulness compared with previous risk scorings. For a 70-year-old sepsis patient with no background of CHF, the following factors were evaluated: INR, 0.83; fibrinogen, 4.87 g/L; CRP, 108 mg/L; SOFA score, 11, with no use of dopamine. The predicted risk of new-onset AF was 33.0% [31]. The analysis by Dai et al. [32] centers on the development of NOAF in sepsis, with a special emphasis on the prognostication of NOAF by inflammatory markers such as myeloperoxidase (MPO) and hypochlorous acid (HOCl). It concluded that septic patients who developed NOAF had worse outcomes in the form of longer durations of hospitalization as well as a higher risk of stroke as well as death. NOAF became strongly associated with enhanced overall inflammation, with higher concentrations of MPO, HOCl, TNF-α, and WBC as prime markers indicating this state of inflammation. These inflammatory markers, particularly those indicating oxidative stress and atrial fibrosis, were considered responsible for AF in sepsis. The analysis named several independent predictors of NOAF, such as higher levels of MPO, HOCl, TNF-α, WBC, and elevated Acute Physiology and Chronic Health Evaluation (APACHE) II scores. Based on these variables, a prediction model accurately identified sepsis patients at risk for developing NOAF. The model offers a useful clinical tool for the early detection of at-risk patients, enabling earlier intervention that can minimize adverse outcomes such as heart failure and stroke [32]. Li et al. investigated the sepsis-induced coagulopathy (SIC) score as a predictor of NOAF among sepsis patients. The researchers found a more excellent SIC score to be a single risk factor for NOAF, indicating that the score can recognize those at high risk to initiate early intervention. The mechanisms behind the association involve enhanced thrombin formation, endothelial dysfunction, and inflammatory responses interfering with cardiac tissue and rhythm. The SIC score assesses important coagulation variables, such as platelet count, prothrombin time, and the SOFA score, and offers a global assessment of coagulation derangement in sepsis. Systemic inflammation, along with coagulation derangement, can, in sepsis, lead to complications such as the development of NOAF, and the SIC score is intended to identify these early. It is especially valuable in detecting at-risk patients for proven complications such as disseminated intravascular coagulation (DIC). Patients were assigned points based on the following criteria: platelet count less than 100 × 10⁹/L (2 points) or between 100 and 150 × 10⁹/L (1 point); D-dimer or fibrin degradation products with a substantial increase (3 points) or a moderate rise (2 points); prothrombin time ratio greater than 1.4 (2 points) or equal to 1 (1 point); fibrinogen level below 100 g/mL (1 point); and a SOFA score of 2 or higher (2 points) or a score of 1 (1 point). A SIC score of 4 or greater indicates a high risk of sepsis-induced coagulopathy. It assists in identifying those at a greater risk of poor outcomes, including the development of NOAF. The score is, therefore, a sound, practical tool for the clinician's early identification of those at risk, enabling them to institute timely management to minimize the occurrence of NOAF and other complications [33]. Many studies pointed out the rising occurrence of atrial ectopic beats before paroxysmal atrial fibrillation (PAF) episodes, which shows they can be used as predictors of primary AF. Additionally, Bashar et al. created a novel automated system focused on predicting the onset of AF among critically ill sepsis patients. The system used the RR interval variation of electrocardiogram (ECG) signals and machine learning classifiers like support vector machine (SVM) and random forest (RF). Using five-minute ECG recordings for feature extraction, the system obtained remarkable findings with 90% accuracy utilizing the SVM model and 88% with the RF model. The tool remarkably predicted AF events at 80% accuracy 10 minutes before their occurrence [34]. The study titled "Predictors of New-Onset AF Burden in the Critically Ill" by Lancini et al. investigates the factors contributing to the burden of NOAF in critically ill patients, with a specific emphasis on left atrial size and underlying chronic cardiovascular conditions. Left atrial size emerged as a significant independent predictor of AF burden, indicating that structural alterations in the heart, particularly atrial enlargement, play a key role in the extent of AF experienced by patients [35]. Similarly, left and right atrial strains (LASr and RASr) were identified as strong predictors of new-onset atrial fibrillation (NOAF) in septic shock patients [36].

**Table 1:** Summary of Predictive Models SAFE score: Atrial Fibrillation in Sepsis score; SIC score: sepsis-induced coagulopathy score; APACHE II score: Acute Physiology and Chronic Health Evaluation II score; SOFA score: Sequential Organ Failure Assessment score; INR: international normalized ratio; CRP: C-reactive protein; FiO_2_: fraction of inspired oxygen; CHF: congestive heart failure; TNF: tumor necrosis factor; WBC: white blood cell count; SVM: support vector machine; RF: random forest; MPO: myeloperoxidase; HOCl: hypochlorous acid; APTT: activated partial thromboplastin time; PT: prothrombin time.

Prediction Model	Study	Predictive Factors Used	Study Type
SAFE Score	Klein Klouwenberg et al. [1]	Age, BMI, immunocompromised status, septic shock status, vasopressor/inotrope use, CRP, WBC, renal failure, potassium, FiO₂	Retrospective cohort study
Coagulation Predictors	Long et al. [30]	Platelet count, INR, APTT (early coagulation abnormalities)	Retrospective cohort study
Nomogram Model	Li Z et al. [31]	Age, INR, fibrinogen, CRP, SOFA score, CHF, dopamine use	Retrospective cohort study
MPO and HOCl Model	Dai et al. [32]	MPO, HOCl, TNF-α, WBC, APACHE II score	Case-control study
SIC Score	Li et al. [33]	Platelets, PT, D-dimer, fibrinogen, SOFA score	Retrospective cohort study
ECG-based Machine Learning Model	Bashar et al. [34]	RR interval variability via ECG; SVM and RF classifiers	Retrospective machine learning model development study
Left Atrial Size Model	Lancini et al. [35]	Left atrial size	Retrospective cohort study
Left and Right Atrial Strain Model	Beyls et al. [36]	Left and right atrial strain	Retrospective cohort study

Developing predictive models for NOAF in sepsis might improve outcomes for this high-risk group. Early identification of at-risk patients allows for possible timely interventions, and the models discussed in this review show strong potential in enhancing clinical decision-making. Coagulation disorders, often signaling the progression of sepsis, may serve as early indicators for preventing life-threatening arrhythmias like AF. Notably, the two nomograms highlighted in this review demonstrated superior calibration and clinical utility, offering clinicians tools to tailor treatment strategies for high-risk individuals. Nonetheless, the generalizability of such models to varied clinical environments is unclear and needs validation. Furthermore, although they are successful at predicting risk, their direct effect on decreasing AF incidence or enhancing survival remains to be proven. Structural and functional characteristics of the atria are shown to be involved in the development of NOAF in critically ill patients, especially those with sepsis and septic shock. Atrial enlargement of the left atrium is the major independent predictor of the burden of AF, as it shows that atrial enlargement is a cause of the initiation and severity of AF. Clinicians can include speckle tracking echocardiography (STE) as a standard practice for evaluating left and right atrial strain. This method offers a valuable understanding of atrial function, allowing for early detection and possible intervention in high-risk populations developing NOAF. Although the potential of predictive models exists, issues crop up concerning the prevention and management of sepsis-induced NOAF. While several predictive models have shown promising performance, many share common limitations. These include dependence on laboratory biomarkers that may not be readily available and have limited external validation. Most models remain at an early stage of evaluation, and caution is warranted when applying them in clinical settings. At present, predictive awareness alone may support closer monitoring but does not yet justify prophylactic interventions. Future predictive models may benefit from combining mechanical predictors such as atrial size or strain with biochemical markers like CRP or MPO. An integrated approach could enhance predictive accuracy by capturing both structural vulnerability and inflammatory burden.

Prevention

Prevention is also a largely untapped strategy for sepsis-induced AF. Early intervention on its causal mechanisms could significantly improve outcomes because of AF's frequency and possible complications during sepsis. Because sepsis itself is a primary cause of AF, the most critical preventive measure may be the optimization of sepsis. Strategies involve minimizing vasopressor overuse, which can worsen sympathetic overactivation, and restricting aggressive fluid resuscitation, which can potentially cause atrial overload and atrial stretch, both factors that promote AF. Preserving electrolyte equilibrium, especially of normal potassium and magnesium levels, is also necessary for cardiac stabilization [9,10]. Prevention may involve a role in anti-inflammatory measures because of the predominant inflammatory etiology of sepsis-induced AF. Glucocorticoids, for example, decreased the incidence of AF by modulating the inflammatory process and reducing the duration of septic shock, thus minimizing catecholamine exposure. Glucocorticoids' effectiveness in sepsis-induced AF prevention has been tested in multiple studies with evidence indicating a possible protective effect. Their anti-inflammatory effects prevent myocardial inflammation, a central mechanism of AF pathogenesis, and decrease septic shock duration, with subsequent diminished vasopressor requirements and lower catecholamine levels, which are factors in preventing AF [18]. In a retrospective analysis of 109 ICU septic shock patients, the use of hydrocortisone (HC) correlated with a statistically lower incidence of AF (20.5% vs. 42.9%), further indicating its protective role [37]. Similarly, a prospective observational study conducted across five academic ICUs in France evaluated the impact of low-dose hydrocortisone in 261 septic shock patients. The overall incidence of AF was 22%. After adjusting for confounders, hydrocortisone significantly reduced AF risk, with a weighted analysis showing an absolute AF risk reduction of 11.9% [38]. A comprehensive analysis of multiple randomized controlled trials has evaluated pharmacologic strategies for preventing supraventricular arrhythmias (SVAs) and AF following cardiac surgery. While the mechanisms and pathophysiology of AF after cardiac surgery differ from sepsis-induced AF, existing evidence on perioperative AF prevention strategies may provide valuable insights for future research on preventing AF in sepsis. A meta-analysis of 24 trials found that beta-blockers significantly reduced SVA incidence after coronary artery bypass graft (CABG) surgery [39]. Similarly, a systematic review of 19 randomized trials demonstrated that amiodarone effectively lowered the risk of AF, ventricular tachyarrhythmias, and stroke following cardiac surgery [40]. Additionally, a meta-analysis of 20 trials involving 2,490 patients assessed the impact of magnesium administration on postoperative AF. The findings showed that magnesium reduced AF incidence from 28% to 18%, indicating its potential role in postoperative arrhythmia prevention [41]. In a meta-analysis by Zhao et al., enrolling a total of 2,031 patients, the effectiveness of colchicine in preventing AF following cardiothoracic surgery or cardiac intervention was investigated. The results revealed that colchicine significantly reduces the incidence of postoperative atrial fibrillation (POAF) in patients undergoing cardiac surgery but not in those undergoing thoracic surgery. While colchicine use was associated with a higher risk of gastrointestinal side effects, it did not significantly impact infection rates or the length of hospital stay. The effectiveness of colchicine is likely attributed to its anti-inflammatory properties, as demonstrated by its ability to reduce CRP and interleukin-6 (IL-6) levels in postoperative patients [42].

**Figure 1:**
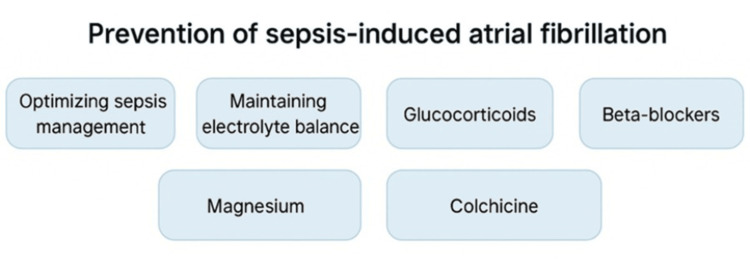
Potential Prevention Strategies for Sepsis-induced Atrial Fibrillation

Since inflammation is a key driver of sepsis-induced AF, anti-inflammatory therapies such as glucocorticoids may offer protection against AF in septic patients. With their potent anti-inflammatory effects, glucocorticoids may help reduce the burden of complications associated with this arrhythmia. Further research on their role in AF prevention could provide valuable insights into improving outcomes for critically ill patients. Although parallels are often drawn between postoperative AF and sepsis-induced AF due to shared inflammatory mechanisms, differences in underlying pathophysiology and patient profiles may limit direct generalizability of prevention strategies. Magnesium is thought to reduce the risk of AF by stabilizing myocardial cell membranes and correcting electrolyte imbalances common in sepsis. Beta-blockers may offer benefit through autonomic modulation, reducing sympathetic overactivity that contributes to atrial electrical instability. Beta-blockers, amiodarone, and magnesium have also shown effectiveness in reducing atrial arrhythmias after cardiac surgery, and their potential role in preventing sepsis-induced AF warrants further investigation. Also, colchicine, a powerful anti-inflammatory medication, has been reported to decrease the risk for AF following surgery by decreasing CRP and IL-6 levels. Since inflammation plays a part in sepsis-induced AF, exploring colchicine to prevent AF among sepsis patients is a promising avenue for continued study. Caution is warranted, particularly in patients with renal or hepatic dysfunction and elevated inflammatory burden, where safety and tolerability require further evaluation. These preventive strategies remain investigational and are not currently part of established sepsis management guidelines.

Management

Sustaining hemodynamic stability is very important in sepsis-related AF, particularly in vasopressor-dependent individuals. It is essential to understand the effect of NOAF on hemodynamic instability to guide management. The primary treatment strategies include anticoagulation for stroke prevention, rhythm control, and rate control. The role of anticoagulation has been discussed earlier. Studies suggest restoring sinus rhythm in sepsis-induced NOAF is associated with lower mortality than patients whose cardioversion was unsuccessful [3,43]. Electrical cardioversion has shown limited efficacy, high recurrence rates, and uncertain survival benefits. While it may restore sinus rhythm temporarily, recurrent AF is common due to the underlying critical illness. Antiarrhythmic medications or electrolyte optimization may improve success rates, but further research is needed. A meta-analysis of multiple studies, including randomized controlled trials (RCTs), identified amiodarone as the most studied drug for rhythm control in sepsis-induced AF. Its success rates vary (18%-94%), with better outcomes observed when administered as a bolus followed by continuous infusion. Other medications, including magnesium, beta-blockers, calcium channel blockers (CCBs), and digoxin, have demonstrated similar effectiveness. Two RCTs and observational data indicate that esmolol is more effective than diltiazem and amiodarone for cardioversion in sepsis-induced AF [44,45]. Magnesium is particularly promising as it stabilizes cardiac conduction without causing hypotension, a common side effect of beta-blockers, calcium channel blockers (CCBs), and amiodarone. The combination of magnesium and amiodarone has shown encouraging results, suggesting a potential benefit for treatment [46]. Managing heart rate (HR) in sepsis-induced AF is challenging, especially in patients receiving vasopressors with chronotropic activity. Despite these challenges, several pharmacological agents have proven effective. Amiodarone and diltiazem significantly reduce HR, demonstrating greater efficacy than metoprolol. Beta-blockers (esmolol and landiolol) show comparable effectiveness to amiodarone and diltiazem, as supported by both randomized and observational studies. Digoxin has also proven effective in ICU settings, achieving rapid and sustained HR control while maintaining stable hemodynamics. Even when cardioversion fails, high-dose amiodarone remains a viable option for rate reduction [44]. A study by Bosch et al. found that beta-blockers achieved the fastest HR reduction within the first hour in patients with sepsis-induced NOAF with rapid ventricular response (RVR). However, all medications were similarly effective by six hours, with amiodarone and CCBs providing better long-term control [47]. Additionally, Law et al. reported that phenylephrine modestly lowered HR compared to norepinephrine in sepsis patients with AF, but its impact on clinical outcomes remains uncertain [48]. Due to variability in HR control definitions, no single pharmacological agent can be universally recommended. However, ultra-short-acting beta-blockers like esmolol and landiolol offer key advantages, including rapid onset, precise titration, and a short half-life, allowing for controlled HR reduction with minimal prolonged beta-blockade risks. Landiolol stands out due to its potent HR-lowering effects and lower hypotension risk, making it particularly suitable for ICU patients. Studies confirm its effectiveness in sepsis-related tachyarrhythmias, enhancing hemodynamic stability and arterial function [44,49,50]. A randomized controlled trial by Kakihana et al. evaluated landiolol in critically ill patients with sepsis-related tachyarrhythmias requiring catecholamine support. Patients received either standard sepsis therapy alone or with landiolol. The study found that 55% of landiolol-treated patients achieved target HR control (60-94 bpm) within 24 hours, compared to 33% in the control group. Landiolol also reduced the incidence of new-onset arrhythmias, demonstrating superior HR stabilization. While generally well-tolerated, careful monitoring for hypotension remains essential in septic patients [51]. Despite numerous studies evaluating rhythm and rate control strategies in sepsis-induced NOAF, there is no established standard of care or widely accepted guidelines for its management. Electrocardioversion shows limited efficacy with high recurrence rates. While pharmacologic interventions such as amiodarone, beta-blockers, and magnesium have demonstrated effectiveness, their long-term impact on clinical outcomes remains uncertain. Using ultra-short-acting beta-blockers like esmolol and landiolol presents a promising approach, particularly in critically ill patients, but further validation is needed. Overall, there is a significant gap in knowledge regarding optimal rhythm and rate control strategies in sepsis-induced NOAF. The current evidence is limited, and randomized clinical trials are urgently needed to determine the most effective and safest approach for managing these patients [44,52,53]. While several agents are frequently used in managing sepsis-induced AF, their use in critically ill patients requires caution due to risks such as bradycardia, hypotension, potential organ toxicity, and other adverse effects. Given the potential impact of NOAF on hemodynamic stability and clinical outcomes, establishing evidence-based management strategies should be a research priority.

## Conclusions

Sepsis-induced NOAF is a clinically significant complication associated with increased risks of stroke, mortality, and recurrence of arrhythmia. While current evidence supports the role of inflammation, hemodynamic stress, and atrial remodeling in its pathogenesis, preventive and management strategies remain largely investigational. Therapies such as beta-blockers, amiodarone, and magnesium show promise, but their application must be carefully individualized due to limited data and safety concerns in critically ill patients. Anticoagulation decisions remain particularly complex, with uncertainty applying both to the acute phase and long-term management post-discharge. Existing stroke risk tools like CHA₂DS₂-VASc have limited validation in this population, stressing the need for improved predictive models that integrate clinical, echocardiographic, and inflammatory markers. The most pressing research gaps include determining the efficacy and safety of anticoagulation, outcomes of rhythm versus rate control strategies, and the long-term consequences of sepsis-induced AF. Ultimately, the development of standardized, evidence-based clinical guidelines should be a key priority to improve outcomes and guide decision-making in this high-risk setting.
